# Identification and characterization of a novel alkalistable and salt‐tolerant esterase from the deep‐sea hydrothermal vent of the East Pacific Rise

**DOI:** 10.1002/mbo3.601

**Published:** 2018-03-05

**Authors:** Xinwei Yang, Lianzuan Wu, Ying Xu, Chongrong Ke, Fangfang Hu, Xiang Xiao, Jianzhong Huang

**Affiliations:** ^1^ National Engineering Research Center of Industrial Microbiology and Fermentation Technology College of Life Sciences Fujian Normal University Fuzhou Fujian China; ^2^ State Key Laboratory of Microbial Metabolism School of Life Sciences and Biotechnology Shanghai Jiao Tong University Shanghai China

**Keywords:** alkalistable, deep‐sea hydrothermal vents, esterase, metagenomic sequence‐based strategy, salt‐tolerant

## Abstract

A novel esterase gene selected from metagenomic sequences of deep‐sea hydrothermal vents was successfully expressed in *Escherichia coli*. The recombinant protein (est‐OKK), which belongs to the lipolytic enzyme family V, exhibited high activity toward pNP‐esters with short acyl chains and especially p‐nitrophenyl butyrate. Site‐mutagenesis results confirmed that est‐OKK contains the nonclassical catalytic tetrad predicted by alignment and computational modeling. The est‐OKK protein is a moderately thermophilic enzyme that is relatively thermostable, and highly salt‐tolerant, which remained stable in 3 mol/L NaCl for 6 hr. The est‐OKK protein showed the considerable alkalistability, displayed optimal activity at pH 9.0 and maintained approximately 70% of its residual activity after incubation at pH 10 for 4 hr. Furthermore, the est‐OKK activity was strongly resistant to a variety of metal ions such as Co^2+^, Zn^2+^, Fe^2+^, Na^+^, and K^+^; nonionic detergents such as Tween‐20, Tween‐80; and organic solvents such as acetone and isopropanol. Taken together, the novel esterase with unique characteristics may give us a new insight into the family V of lipolytic enzymes, and could be a highly valuable candidate for biotechnological applications such as organic synthesis reactions or food and pharmaceutical industries.

## 1 INTRODUCTION

Lipolytic enzymes that catalyze the hydrolysis and synthesis of esters belong to one of the most important groups of biocatalysts for biotechnological applications such as the synthesis of fine chemicals or those found in the food and pharmaceutical industries (Bornscheuer, 2002). Esterases (EC 3.1.1.1) and lipases (EC 3.1.1.3) are two major types of lipolytic enzymes. The former (EC 3.1.1.1) hydrolyzes water‐soluble short‐chain fatty acid esters (C < 10), whereas the latter prefer water‐insoluble long‐chain triglycerides (C > 10) (Jaeger, Dijkstra, & Reetz, 1999). Both esterases and lipases possess a characteristic α/β hydrolase fold that includes a consensus sequence (Gly‐x‐Ser‐x‐Gly) and a conserved catalytic triad (Ser‐Asp/Glu‐His) (Arpigny & Jaeger, 1999; Nardini & Dijkstra, 1999).

Deep‐sea hydrothermal vents are an extreme environment that is full of extremophiles such as thermophiles, hyperthermophiles, halophiles, alkaliphiles/acidophiles, and solvent‐resistant microorganisms. There is no doubt that extremozymes from extremophiles have become increasingly attractive for modern biotechnology due to their excellent performance under extreme physico‐chemical conditions (Elleuche, Schroder, Sahm, & Antranikian, 2014). However, discovering new genes using traditional separation methods is very difficult (Schloss & Handelsman, 2003). Metagenomics, a cultivation‐independent method, provides a way to discover new functional genes in both cultured and uncultured microorganisms. Since the potential of functional metagenomics was first discovered by Rondon, Raffel, Goodman, & Handelsman (1999), various enzymes such as carboxylesterase (Alcaide, Stogios, et al., 2015; Alcaide, Tchigvintsev, et al., 2015; Popovic et al., 2017), beta‐glycosidase (Matsuzawa & Yaoi, 2017), cellulase (Garg et al., 2016; Zhao et al., 2017) and chitinase (Berini et al., 2017) have been identified using metagenomic screening.

Lipolytic enzymes that were discovered by metagenomic analysis represent a hot direction for the identification of novel enzymes for technological application. A series of novel esterases have been isolated from metagenomic libraries of different habitats such as deep‐sea (Alcaide, Stogios, et al., 2015; Alcaide, Tchigvintsev, et al., 2015; Fu et al., 2011; Zhang et al., 2017), neritic sediments (Peng et al., 2011), marine arctic (De Santi et al., 2016) and tidal flat sediments (De Santi et al., 2016; Jeon et al., 2012). However, lipolytic enzymes that were isolated from the habitats of deep‐sea hydrothermal vents are very limited. Zhu et al. (2013) identified a thermostable esterase, EstEP16, from a deep‐sea hydrothermal field in the eastern Pacific that retained approximately 80% of its residual activity after incubation at 90°C for 6 hr. Fu et al. (2015) detected a thermostable patatin‐like esterase by functionally screening fosmid environmental DNA libraries from a Guayas Basin smoker. Placido et al. (2015) selected a series of lipases/esterases from hydrothermal vent sediments of the Levante Bay at Vulcano Island. Among them, the LIPESV12_24 was the most temperature adapted and active in organic solvents.

The environment of deep‐sea hydrothermal vents has some characteristic features: high‐temperature, abundant metal ions, reduced amount of gases, salinity, the presence of a variety of electron donors and acceptors and diverse microbial metabolisms, which makes them a valuable site for metagenomic enzyme study. In this work, we selected an esterase gene from a deep‐sea hydrothermal vent at 9°50′N site of the East Pacific Rise using a metagenomic sequence‐based strategy. A novel esterase (est‐OKK) that exhibited robust activity in an alkaline environment and tolerance to salts, organic solvents, and metal ions was successfully identified, expressed, and characterized. Our results prove that metagenomics is a useful tool in mining novel enzymes with extreme features, and that with the metagenomic sequence‐based strategy, the secret and valuable resources in deep‐sea hydrothermal vents can be easily detected and applied to industrial biotechnology.

## 2 MATERIALS AND METHODS

### 2.1 Strains, plasmids, and chemicals

The plasmid pET‐28a(+) was used to express the target gene in the expression host *Escherichia coli* BL21(DE3). Restriction endonucleases, DNA polymerase, and T4 DNA ligase were purchased from NEB (New England BioLabs, China). Nickel columns were purchased from GE (General Electric Company, China). p‐Nitrophenyl esters were obtained from Sigma (St. Louis, USA). All other chemicals were of analytical grade and purchased from Sangon (Shanghai, China).

### 2.2 Lipolytic gene screening

Sediment samples were collected from a deep‐sea hydrothermal vent at 9°50′N site of the East Pacific Rise. The metagenomic dataset of the sediment was generated by pyro‐sequencing by Laboratory of Microbial Oceanography of Shanghai Jiao Tong University (unpublished data). All metagenomic sequences were functionally annotated using the Clusters of Orthologous Groups (COGs) of proteins database. COGs were identified by BLASTX analysis with an *E*‐value cut‐off of 1e^−5^ (Tatusov et al. 2000), and the sequences were assigned to COG functional categories based on their best BLAST hits. COG2267 was chosen for further analysis, and the *est‐okk* gene was synthesized and codon‐optimized based on the amino acid sequence from metagenomic sequences (GenBank accession MF277135).

### 2.3 Bioinformatic analysis of lipolytic gene

Analysis of the amino acid composition of the est‐OKK was performed using the Protein BLAST program at the NCBI website. The potential signal peptide sequence was predicated by SignalP4.0 (http://www.cbs.dtu.dk/services/SignalP/). Multiple sequence alignment was carried out with clusterW (http://www.ebi.ac.uk/Tools/clustalw2/) and ESPript3.0 (Robert & Gouet, 2014). A phylogenetic tree, including the est‐OKK and other lipolytic families, was constructed using the neighbor‐joining method with MEGA 5.0 (Tamura, Dudley, Nei, & Kumar, 2007). The CPHmodel was used to predict the three‐dimensional structure of the est‐OKK (Nielsen, Lundegaard, Lund, & Petersen, 2010). Analysis of the homology model was evaluated by PROCHECK (Laskowski, Rullmannn, MacArthur, Kaptein, & Thornton, 1996). The predicted model and surface electrostatic potential were visualized via VMD with the assistance of APBS plugin (Unni et al., 2011).

### 2.4 Expression and purification of the enzyme

The constructed plasmid pET‐28a‐OKK was transformed into *E. coli* BL21 (DE3). The transformants were grown in LB broth that contained 50 μg/ml of kanamycin for 10 hr at 37°C with rotary agitation at 220 rpm. When the OD_600_ reached 0.6, 0.5 mmol/L isopropyl‐β‐D‐thiogalactopyranoside (IPTG) was added to induce protein expression at 20°C for 16 hr. After incubation, the cells were harvested, resuspended in phosphate buffer (50 mmol/L PBS containing 20 mmol/L imidazole) and disrupted by sonication on ice. The recombinant His‐tagged protein in the extract was purified by Ni affinity chromatography (AKTAprime plus, GE, USA) and then dialyzed in phosphate buffer (50 mmol/L PBS, pH 8.0) overnight to remove imidazole using a microfilter (Micro‐con YM‐10, Millipore Corp, USA). Protein concentrations were determined using the Bradford protein assay (Modified Bradford Protein Assay Kit, Sangon biotech, CN) with bovine serum albumin as a standard.

### 2.5 Enzymatic Activity Assay

The esterase activity was determined by monitoring the hydrolysis of p‐nitrophenyl esters using a spectrophotometric method that involved measuring the optical density (OD) at 410 nm. The standard reaction mixture, which contained 100 μL of various substrates (p‐nitrophenyl acetate (pNP‐A), p‐nitrophenyl butyrate (pNP‐B), p‐nitrophenyl caprate (pNP‐C), p‐nitrophenyl octanoate (pNP‐O), p‐nitrophenyl dodecanoate (pNP‐D), p‐nitrophenyl palmitate (pNP‐P), and p‐nitrophenyl myristate (pNP‐M)), 2,870 μL of reaction buffer (50 mmol/L Tris‐HCl pH 9.0) and 30 μL of diluted protein (concentration of 8 μg/ml), was incubated at 50°C for 5 min. One unit of enzyme activity was defined as the amount of enzyme that released 1 μmol of p‐nitrophenol per minute under the above conditions. To eliminate the influence of substrate self‐decomposition, a solution with reaction buffer but lacking enzyme was used as a control. All reactions were performed in triplicate.

### 2.6 Enzyme characterization

The optimum temperature of the purified est‐OKK activity was determined using pNP‐B as a substrate over a range from 20°C to 60°C in Tris‐HCl buffer (pH 9.0). The thermostability of the purified est‐OKK was determined by measuring the residual activity after incubating the enzyme solution at 55°C and 65°C for 30, 60, 90, and 120 min, and then quickly cooled down on the ice. The optimal pH of the esterase activity of purified est‐OKK was determined at 50°C in buffers with pH values that ranged from 6.0 to 11.0. The buffers with different pH values were 50 mmol/L CH_3_COOH‐CH_3_COONa for pH 4.0–6.0, 50 mmol/L NaH_2_PO_4_‐Na_2_HPO_4_ for pH 6.0–8.0, 50 mmol/L Tris‐HCl for pH 8.0–9.0, and 50 mmol/L NaOH‐glycine for pH 9.0–11.0. pH stability was determined by incubating the enzyme in Tris‐HCl (pH 9.0) and NaOH‐glycine (pH 10.0) buffers for 1, 2, 3, and 4 hr, and the remaining activity was measured by the standard method described above.

The effect of NaCl on the purified est‐OKK activity was determined at 50°C in Tris‐HCl buffer (pH 9.0) that contained 0–3.0 mol/L NaCl. To examine its resistance to salt, est‐OKK was incubated in 0–3.0 mol/L NaCl at room temperature for 4 hr, and the residual enzyme activity was measured.

The kinetic parameters of the purified est‐OKK were measured in Tris‐HCl buffer (pH 7.5) that contained different concentrations (0.1, 0.2, 0.4, 0.5, 0.6, and 0.7 mmol/L) of pNP‐B at 50°C for 5 min. The Michaelis constant (*K*_m_) and maximum activity (*V*_max_) were calculated from Michaelis‐Menten equation using the nonlinear curve fitting function in OriginPro 8.5.

The effects of various compounds on purified est‐OKK were determined by measuring the activity after the addition of 2 or 10 mmol/L metal ions (Ni^2+^, Al^3+^, Mn^2+^, Fe^3+^, Co^2+^, Cu^2+^, Mg^2+^, Ca^2+^, Fe^2+^, Zn^2+^, K^+^, and Na^+^), 1% surfactants (Tween20, Tween80, Triton X‐100 and SDS) and potential inhibitors (EDTA, PMSF, DTT, GSH and urea) to the reaction buffer. The impact of organic solvents on the est‐OKK activity was determined by measuring the residual activity after incubating the enzyme with 20% (v/v) of the different organic solvents (methanol, acetone, ethanol, isopropanol, and acetonitrile).

## 3 RESULTS

### 3.1 Sequence and phylogenetic analysis of est‐OKK

A metagenome that was isolated from a deep‐sea hydrothermal vent sample was sequenced and screened into COG functional categories. Among the sequences that were assigned was COG2267; this est‐OKK gene is 804 bp long and was predicted to encode a lipolytic enzyme, leading us to choose it for further analysis. The est‐OKK gene encoded a lipolytic enzyme of 268 amino acids with a predicted molecular mass of 33 kDa and a predicted pI of 7.95. SignalP results suggested that no N‐terminal signal peptide was present in the whole gene.

Phylogenetic tree based on 36 classical lipolytic enzymes showed that est‐OKK belongs to the same branch of five previously described members of family V of bacterial lipolytic enzymes; these members are the lipase from *Moraxella* sp. (CAA37863), the lipase from *Psychrobacter immobilis* (CAA47949), the putative esterase from *Haemophilus influenzae* (AAC21862), the lipase from *Fervidobacterium changbaicum* (ABL95965), and the lipolytic enzyme from *Sulfolobus acidocaldarius* (AAC67392) (Figure S1).

### 3.2 Noncanonical catalytic tetrad of est‐OKK

Alignment of the est‐OKK with four of the representative lipolytic enzymes of family V showed that they possess three similar canonical conserved domains (block I‐III) including consensus pentapeptide GHSXG and an active site (Ser85, Ser214, and His242) (Figure S2). Among them, Ser85 is located in the GXSXG sequence motif in block II, while Ser214 and His242 are located in block III. Unlike the canonical catalytic triad, the Asp residue of est‐OKK is replaced by a Ser residue in block III. Interestingly, the additional conserved site Asp109 was found between β‐sheet 4 and α‐helix 4 (Figure S3). This result suggested that Asp109, together with the other three active sites, may form a catalytic tetrad that is similar to YbfF in *E. coli* (Park et al., 2008).

**Figure 1:**
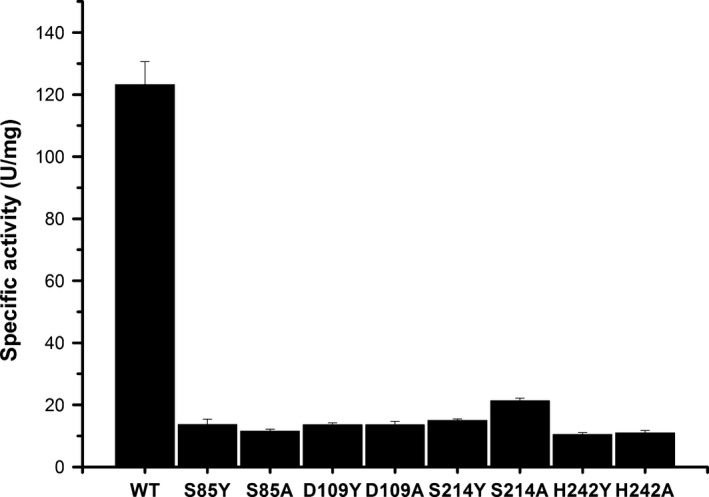
Enzymatic activities of est‐OKK proteins from WT and variants (S85Y, S85A, D109Y, D109A, S214Y, S214A, H242Y, and H242A) after Ni affinity chromatography. Among the mutants of est‐OKK, S85Y represented the eighty‐fifth serine mutation into tyrosine of est‐OKK; Ser, Asp, His, Ala, and Tyr were represented by S, D, H, A, and Y

To test the predicted catalytic tetrad, we selected this four residues (Ser85, Asp109, Ser214, and His242) for site‐directed mutation to investigate their roles in est‐OKK. Hydrophobic amino acids (Tyr and Ala) were chosen to replace the original four residues, which are all hydrophilic amino acids. The est‐OKK mutants S85Y, S85A, D109Y, D109A, S214Y, S214A, H242Y, and H242A purified after Ni affinity chromatography (Figure S4) retained only 9%–16% activity of the wild‐type (WT) est‐OKK (Figure 1). The results confirmed our hypothesis of the catalytic tetrad existed in est‐OKK.

### 3.3 Structural modeling of est‐OKK

**Figure 2:**
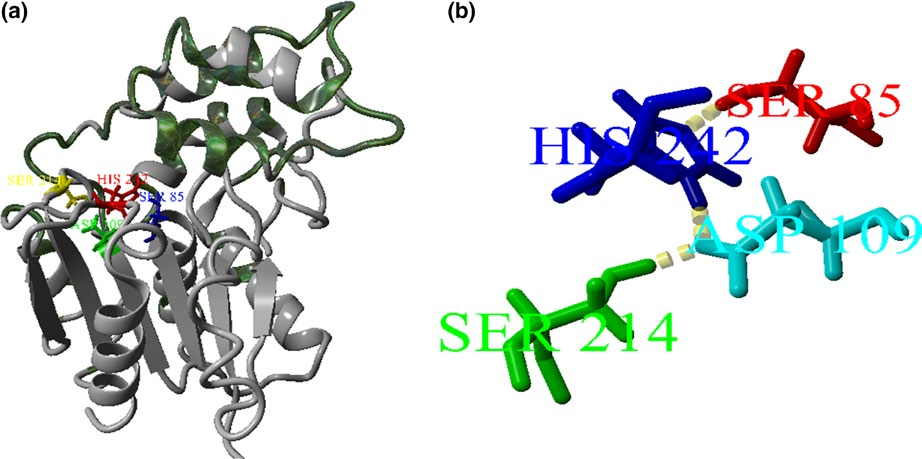
Predicted structure and the catalytic tetrad of est‐OKK. (a) Modeled est‐OKK was constructed with a CPHmodel using 3BF7.B used as the template. The cap structure was represented by dark green color, and the catalytic tetrad (Ser85, Ser214, Asp109, and His242) were represented by blue, yellow, green, and red, respectively. (b) The catalytic tetrad consisted of Ser85, Asp109, Ser214, and His242

A model of est‐OKK was built using CPHmodel based on the template esterase 3BF8.B in the Protein Data Bank (PDB). An analysis of the predicted model by the PROCHECK program showed that over 90% of the residues were located in the reasonable region, indicating that the homology is reliable. The modeled est‐OKK had a structure that was typical of classical α/β hydrolases and consisted of a core domain that was shadowed by a cap (Figure 2a). The core domain contained a three‐layer α/β/α architecture that employed a conserved Ser‐Asp‐Ser‐His catalytic tetrad (Figure 2b). The catalytic active site of Ser85 was located in a groove that is derived from the conserved GXSXG sequence motif in the short β3–α3 loop of the est‐OKK structure. Ser214 interacts with Asp109, which is hydrogen‐bonded to the side chain of His242. This His residue again forms a hydrogen‐bond with another serine, Ser85.

### 3.4 Overexpression and characterization of est‐OKK

**Figure 3:**
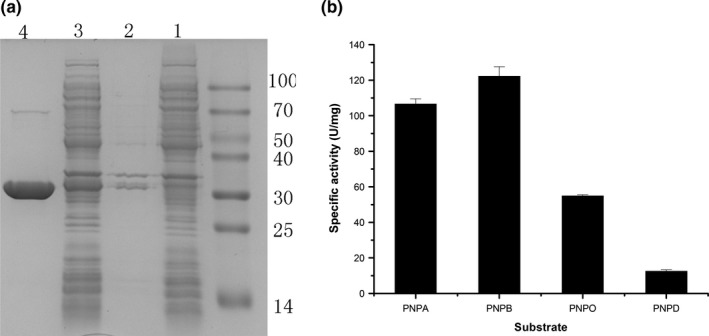
Sodium dodecyl sulfate polyacrylamide gel electrophoresis (SDS‐PAGE) analysis of purified est‐OKK and the substrate specificity of est‐OKK. (a) SDS‐PAGE analysis of purified est‐OKK. Lane 1, soluble extract of *Escherichia coli* cells carrying pET‐28a plasmid; Lane 2, inclusion bodies of the recombinant *E. coli* cells; Lane 3, soluble extract of the recombinant *E. coli* cells; Lane 4, purified est‐OKK from His‐tag affinity chromatography; lane M, protein mass markers. (b) Substrate specificity of est‐OKK, evaluated with pNP esters (PNPA, PNPB, PNPO and PNPD represented the substrate p‐nitrophenyl acetate, p‐nitrophenyl butyrate, p‐nitrophenyl octanoate, p‐nitrophenyl decanoate, respectively). The graph showed data from triplicate experiments (mean ± *SD*)

The *est‐okk* gene was overexpressed in *E. coli* BL21 (DE3), and the recombinant est‐OKK was purified by Ni affinity chromatography. The SDS‐PAGE analysis demonstrated that the est‐OKK was highly pure and displayed an apparent molecular mass of 31.3 kDa, which is consistent with the molecular mass that was calculated from the predicted amino acid sequence (Figure 3a). Furthermore, the purification factor and the purity efficiency of the protein purification process were approximately 8.61% and 22.53% (Table 1), respectively. The purified est‐OKK exhibited 127.3 U/mg of specific activity when pNP‐B was used as a substrate in 50 mmol/L Tris‐HCl (pH 9.0) at 50°C.

**Table 1:** Purification of est‐OKK from *Escherichia coli* BL21 (DE3)

Step	Total protein (mg)	Total activity (U)	Specific activity (U/mg)	Purification fold	Yield (%)
Supernatant of cell lysate	81.90	1,210.48	14.78		
Ni‐NTA agarose affinity	2.14	272.68	127.30	8.61	22.53

To characterize the est‐OKK, pNP esters with different carbon chain lengths (C2, C4, C8, C10, C12, C14, and C16) were used as the hydrolyzing substrates under conditions of pH 9.0 and 50°C. The results revealed that est‐OKK protein could efficiently hydrolyze short‐chain pNP esters (C4–C10), with maximal activity toward p‐nitrophenyl butyrate (Figure 3b). The est‐OKK was unable to catalyze pNP esters longer than 10 carbon atoms (C > 10), indicating that the OKK protein is an esterase. The *K*_m_ and *V*_max_ of est‐OKK against p‐nitrophenyl butyrate at pH 7.5 and 50°C that were obtained by Michaelis‐Menten equation were 502.3 μmol/L and 55.23 U/mg, respectively (Figure S5).

### 3.5 Optimal temperature and thermostability of est‐OKK

**Figure 4:**
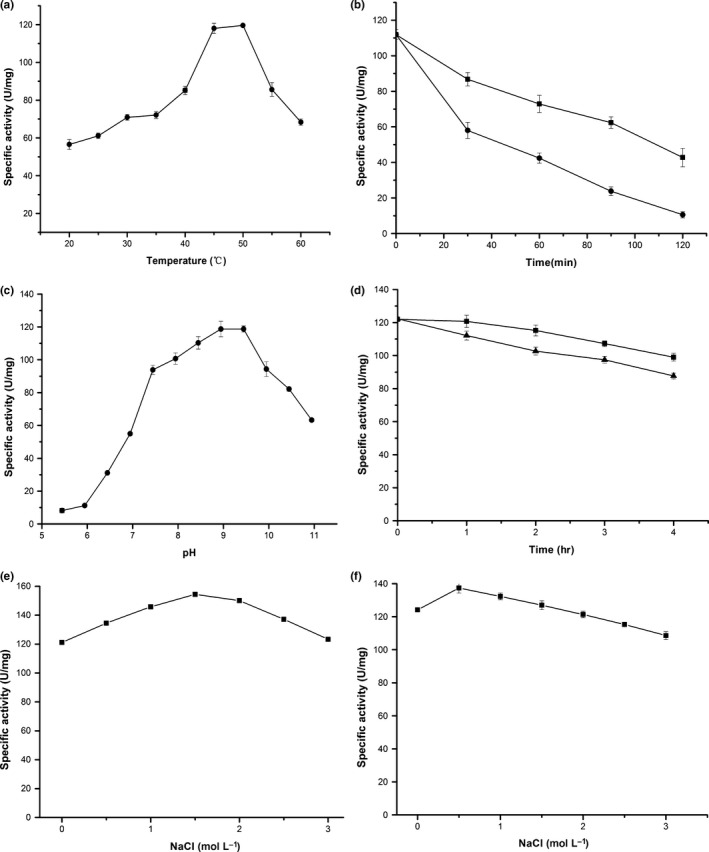
Effect of temperature, pH and NaCl on the activity and stability of est‐OKK. (a) The temperature optimum of est‐OKK. Activities were detected from 20°C to 60°C to identify the optimum temperature of est‐OKK. (b) The thermostability analysis of est‐OKK. The thermostability was determined at 55°C (black square) and 65°C (black circle) by measuring the residual activity under optimal conditions and increasing the incubation time from 30 to 120 min. (c) The pH optimum of the est‐OKK. Activities were detected from pH 6.0 to pH 11.0 to identify the optimum pH of est‐OKK. (d) The alkaline tolerance analysis of est‐OKK. The residual activity was measured under optimal conditions after incubation of est‐OKK at pH 9.0 (black square) and 10.0 (black triangle) for 1–4 hr. (e) Salt‐tolerance of est‐OKK. Activities were detected from 0 mol/L NaCl to 3 mol/L NaCl to identify salt‐tolerance of est‐OKK. (f) Salt stability of est‐OKK was determined by incubating the enzyme with different concentrations of NaCl (0–3 mol/L) for 6 hr and measuring the activity

The activity of the est‐OKK was determined at temperatures ranging from 20°C to 60°C. The est‐OKK protein exhibited its highest activity at 50°C with an ascending tendency from 20°C to 50°C. The est‐OKK protein maintained more than 50% activity at temperatures ranging from 20°C to 60°C, indicating its broad temperature adaptability (Figure 4a). The thermostability of est‐OKK was also determined by detecting the residual activity after incubating the est‐OKK protein at 55°C and 65°C. The est‐OKK enzyme is a thermostable enzyme with a half‐life (*t*_1/2_) that is longer than 90 min at 55°C and longer than 30 min at 65°C (Figure 4b).

### 3.6 Optimal pH and alkalistability of est‐OKK

The activity of est‐OKK was determined at pH values from 6 to 11. The esterase activity had an ascending tendency from pH 6 to 9, exhibiting a particularly sharp increase from 6.5 to 8.0, and then trended downward above pH 9.5. Notably, the est‐OKK showed 50% of its original activity at pH ranging from 8 to 11, indicating that it is an alkaline enzyme (Figure 4c). The alkalistability of est‐OKK was further measured by incubating the enzyme in Tris‐HCl (pH 9.0) and glycine‐NaOH (pH 10.0) buffer. After 4 hr of incubation, est‐OKK still retained 80% of its activity at pH 9.0 and 70% of its activity at pH 10.0. Moreover, est‐OKK maintained 90% of its activity at pH 9.0 for 2 hr and at pH 10.0 for 1 hr (Figure 4d).

### 3.7 High salt tolerance of est‐OKK

Because the est‐OKK gene was isolated from a deep‐sea sediment, we investigated the influence of different concentrations of NaCl on its activity and stability. The activity of est‐OKK was significantly stimulated by salt concentrations from 0.5 to 3 mol/L and peaked between 1.5 and 2 mol/L NaCl with an activity of approximately 120%. The activity of est‐OKK was still maintained at a high level in 3 mol/L NaCl (Figure 4e). Moreover, the enzymatic activity was increased nearly 20% after the enzyme was incubated with different concentrations of NaCl (from 0.5 to 2 mol/L) for 6 hr. When incubated with 2.5 and 3 mol/L NaCl for 6 hr, approximately 92% and 87% of the original activity, respectively, of est‐OKK was retained (Figure 4f). These results suggested that est‐OKK is a halotolerant enzyme.

### 3.8 Effect of metal ions and EDTA on est‐OKK

The impact of various metal ions on the activity of est‐OKK was investigated (Table 2). The activity was slightly stimulated (by 31.7%, 18.8%, 18.4%, 16.7%, 9.4%, and 15.3%) by 2 mmol/L Ni^2+^, Al^3+^, Mn^2+^, Co^2+^, Mg^2+^, and Zn^2+^, respectively. In contrast, its activity was significantly reduced by 2 mmol/L Cu^2+^ or Fe^3+^, in which cases it retained only 46.2% and 26.1% of its activity, respectively. However, when the metal ion concentration of Ni^2+^, Al^3+^, and Mn^2+^ was increased to 10 mmol/L, only 45.2%, 49.4%, and 62.0% of the original activity was retained, indicated that the 10 mmol/L Ni^2+^, Al^3+^, and Mn^2+^ acted as inhibiting factors. Surprisingly, the enzymatic activity could be activated by K^+^, Na^+^, and Zn^2+^, even when their concentrations were increased to 10 mmol/L. Furthermore, the activity of the est‐OKK was not obviously affected by EDTA chelation, suggesting that the enzyme is not a metalloenzyme.

**Table 2:** Effect of metal ions and EDTA on enzyme activity

Compound	Relative activity (%) at concentration of	
	2 mmol/L	10 mmol/L
NONE	100	100
Ni2+	131.7 ± 3	45.2 ± 5
Al3+	118.8 ± 7	49.4 ± 8
Mn2+	118.4 ± 6	62.0 ± 6
Fe3+	46.2 ± 4	7.4 ± 4
Co2+	116.7 ± 6	90.9 ± 3
Cu2+	26.1 ± 5	ND
Mg2+	109.4 ± 5	87.5 ± 5
Ca2+	96.9 ± 3	69.9 ± 9
Fe2+	94.1 ± 1	92.9 ± 5
EDTA	98.6 ± 5	62.8 ± 7
Zn2+	115.3 ± 6	102.9 ± 2
K+	102.3 ± 1	128.9 ± 2
Na+	101.7 ± 3	129 ± 2

### 3.9 Effects of reductants, inhibitors, organic solvents, and detergents on est‐OKK

The impacts of reductants (DTT and GSH) and enzyme inhibitors (PMSF and urea) on the activity and stability of est‐OKK were determined (Table 3). After incubating with GSH and DTT at a concentration of 1 mmol/L, 100.5% and 97.8% of the activity of est‐OKK was maintained, respectively. Significant inhibition was observed in the presence of PMSF at a concentration of 1 mmol/L, resulting in the enzyme activity strongly decreased by 63%. Moreover, only 28% of the enzyme activity was maintained when the enzyme was incubated in 8 mol/L urea, indicating that high concentrations of urea could affect the enzyme fold (Table 3).

**Table 3:** Effect of surfactant agents and reductants on enzyme activity

Compound	Concentration	Relative activity (%) ± *SE*
NONE	–	100
PMSF	(1 mmol/L)	37 ± 2
DTT	(1 mmol/L)	100.5 ± 2
GSH	(1 mmol/L)	97.8 ± 2
Tween‐20	1% (V/V)	98.1 ± 3
Tween‐80	1% (V/V)	97.7 ± 2
Triton X‐100	1% (V/V)	89.96 ± 3
Urea	8 mol/L	28.1 ± 1
SDS	1%	4 ± 1

The est‐OKK protein's resistance against various detergents (Tween 20, Tween 80, Triton X‐100, and SDS) and organic solvents was also determined (Table 4). The nonionic detergents such as Tween 20, Tween 80, and Triton X‐100 have little impact on enzyme activity, while 1% SDS completely inhibited the est‐OKK activity. The enzyme retained more than 70% of its activity after being incubated with a high concentration (20%) of organic solvents (methanol, ethanol, and isopropanol) for 1 hr. Moreover, the enzyme activity was increased 16% when it was incubated with 20% acetone for 1 hr.

**Table 4:** Effect of organic solvents on enzyme stability

Compound	Concentration (%, V/V)	Relative activity (%) ± *SE*
NONE		100
Methanol	20	89.6 ± 2
Ethanol	20	74.1 ± 1
Acetone	20	116 ± 2
Isopropanol	20	98.1 ± 3
Acetonitrile	20	39.9 ± 2

## 4 DISCUSSION

In this work, we characterized a novel esterase from a metagenomic dataset of a deep‐sea hydrothermal vent, which belongs to family V of the bacterial lipolytic enzymes according to the phylogenetic analysis. The members of family V originate from a wide range of bacteria (Prive et al., 2013; Ruiz, Falcocchio, Pastor, Saso, & Diaz, 2007) and share significant homology with other bacterial enzymes such as epoxide hydrolases, dehalogenases, and haloperoxidases (Hausmann & Jaeger, 2010). Among the characterized lipolytic enzymes, est‐OKK shares the highest identity (33%) with YbfF from *E. coli* (GAF03548). Notably, the catalytic tetrad confirmed in est‐OKK structure was similar to that observed in YbfF (Park et al., 2008), which was the first and the only reported esterase with a catalytic tetrad. However, est‐OKK can only efficiently hydrolyze short‐chain pNP‐esters (C2–C10) with highest activity toward pNP‐B, while YbfF had activity toward a wide range of substrates such as palmitoyl‐CoA, malonyl‐CoA, pNP‐B, and several triacylglycerides (Godinho, Reis, Tepper, Poelarends, & Quax, 2011; Park et al., 2008).

The est‐OKK protein was salt tolerant and stimulated by low concentrations of NaCl, reflecting its specific habitat characteristics. Unlike most reported halotolerant/halophilic enzymes that have high acid/base residue ratios and relatively low pI values that range from 4.3 to 6.8 (De Santi et al., 2016; Wang et al., 2016), the number of est‐OKK acidic residues (Asp and Glu, 13.8%) was comparable to the number of basic residues (Arg and Lys, 13.4%) in its sequence (Table S1), leading to an alkalescent pI values. In addition, the analysis of a predicted structure suggested that est‐OKK exhibits a large number of negatively charged acidic residues on the protein surface (Figure S6), which might contribute to a solvation shell that keeps the protein surface hydrated and thus highly tolerant to salt (Danson & Hough, 1997).

In fact, several esterases in family V displayed their highest activity at pH values between 8 and 10; however, very few of them are extremely alkaline stable (pH 10–12) (Cai et al., 2011; Peng et al., 2011; Pereira, Mercaldi, Maester, Balan, & Lemos, 2015; Zhang et al., 2017)). The est‐OKK protein showed its highest activity at pH 9–9.5, retaining over 70% of its highest activity after 4 hr of incubation in buffers at pH 10.0, indicating its potential for industrial use. According to previous reports, an alkalistable enzyme may possess more positively charged amino acids, which are acquired to form ion pairs to maintain the stability of the conformation under high‐alkaline conditions (Shirai et al., 1997). Similar to other alkalistable esterases such as Tm1350 from *Thermotoga maritima* (13.8%) (Tian et al., 2015) and EST‐SL3 from *Alkalibacterium* sp. SL3 (13.7%) (Wang et al., 2016), est‐OKK has a high proportion (13.8%) of positively charged amino acids.

The thermostability feature of est‐OKK was not in line with our expectations because this value is certainly lower than those of other reported thermophilic enzyme such as the esterase LIPESV12_24 or the thermostable lipolytic enzyme PLP from a deep‐sea hydrothermal vent (Fu et al., 2015; Placido et al., 2015). This result indicated that, often, enzymes do not necessarily have to function optimally at the temperature of their environment but merely to function to a degree that gives a physiological advantage. Compared with its thermophilic counterpart, est‐OKK has fewer proline residues (4.5% vs. 6.84% and 10.30%), fewer salt bridges and a lower hydrogen bond percentage than thermophilic EstA and LIPESV12_24 (Levisson et al., 2009; Placido et al., 2015).

Stability and activity in the presence of additives such as organic solvents, detergents, inhibitors and metal ions are important properties of an enzyme if it is to be used as a biocatalyst in the industry. The est‐OKK protein showed normal activity or was slightly activated in 20% of methanol, isopropanol, and acetone, indicating that est‐OKK could serve as a potential reagent in non‐aqueous reactions and organic synthesis. This feature that est‐OKK displayed high activity in the presence of non‐ionic detergents such as Tween‐20, Tween‐80, and Triton X‐100, together with alkalistability, might be useful in the washing or laundry industries. Most of the metal ions mainly activated est‐OKK or had little influence at a low concentration, except that 2 mmol/L Fe^3+^ and 2 mmol/L Cu^2+^ would inhibit its activity, indicating that est‐OKK is derived from a reducing environment. In contrast, 2 mmol/L Ni^2+^ and 10 mmol/L K^+^ or Na^+^ promoted the activity of OKK by 30%, indicating that these metal ions would promote the reaction when est‐OKK was used to hydrolyze or synthesize esters.

## CONFLICT OF INTEREST

The authors declare that they have no conflict of interest.
