# Supplementary material for: Identification and characterization of a novel alkalistable and salt‐tolerant esterase from the deep‐sea hydrothermal vent of the East Pacific Rise
Source: Microbiologyopen. 2018 Mar 5;7(5):e00601. doi: 10.1002/mbo3.601 (PMC6182558; doi:10.1002/mbo3.601)
Supplement: Supplementary file 1 [file MBO3-7-e00601-s001.docx]

**Supporting Information**

**Table S1** Comparison of the amino acid compositions of est-OKK and other salt-tolerant esterases

| Esterase | Composition (%) | | | | Relative activity (%)  with NaCl (M) | Reference |
| --- | --- | --- | --- | --- | --- | --- |
|  | Charged amino  acids | Acidic amino  acids | Hydrophobic  amino acids | |  |  |
| est-OKK | 30.9 | 13.4 | | 34.1 | 113.2 (2.5) | This study |
| ESTKT4 | 25.6 | 12.5 | | 35.1 | 110 (1) | [2] |
| Est12 | 19.7 | 8.8 | | 32.8 | 115 (4.5) | [4] |
| Est9X | 19.3 | 8.2 | | 38.1 | 190 (4) | [1] |
| Est10 | 21.7 | 11.2 | | 37.7 | 140 (2) | [3] |
| EstPc | 24.7 | 11.4 | | 37.9 | 183.4 (1.5) | [5] |


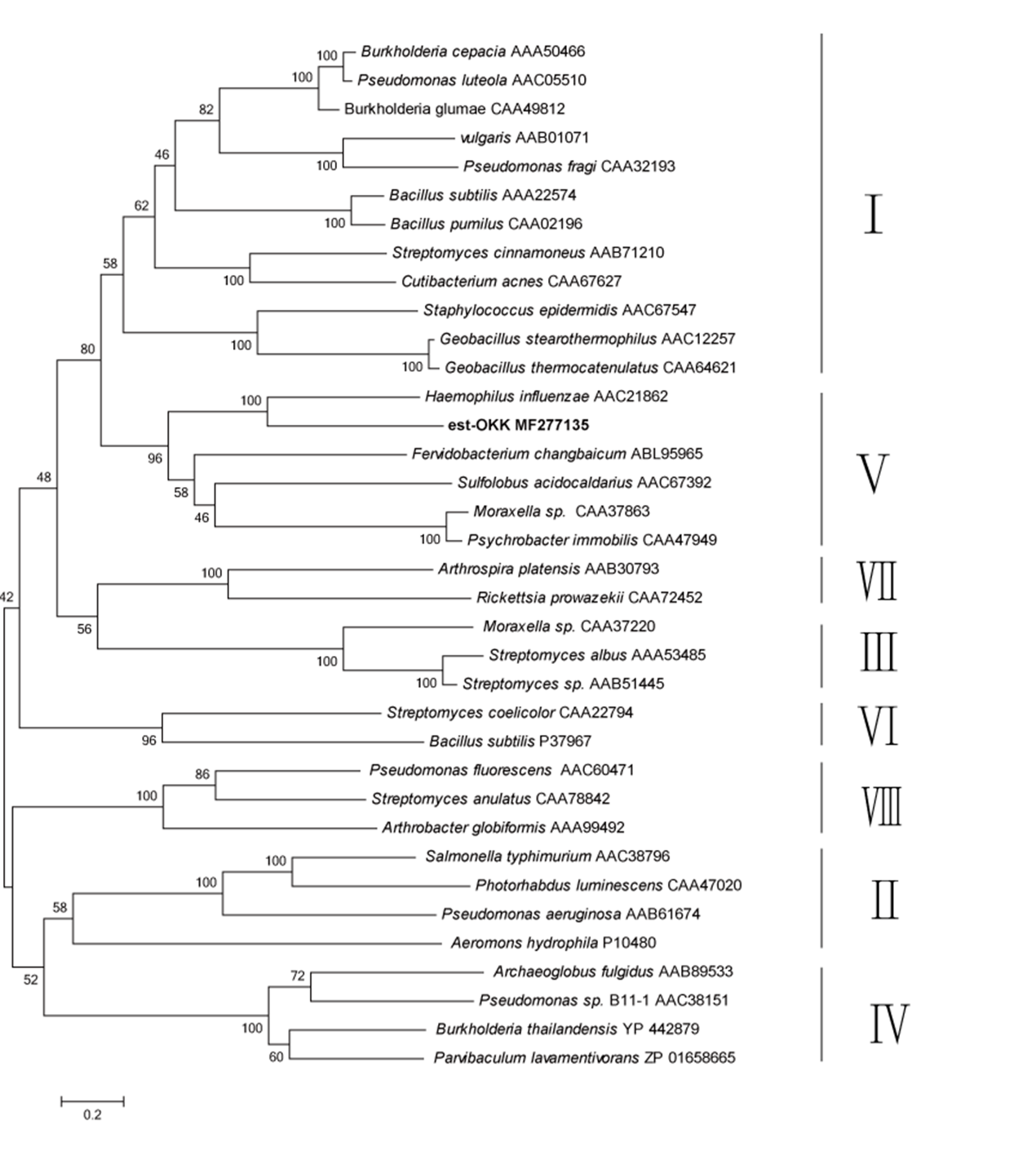


**Figure S1** Phylogenetic tree of lipolytic enzymes based on the neighbor-joining method (bootstrap values based on 1000 replication), constructed using MEGA 5.0 software. The sequences that were used in this analysis were obtained from the GenBank database. The scale bar represents the number of changes per amino acid position.


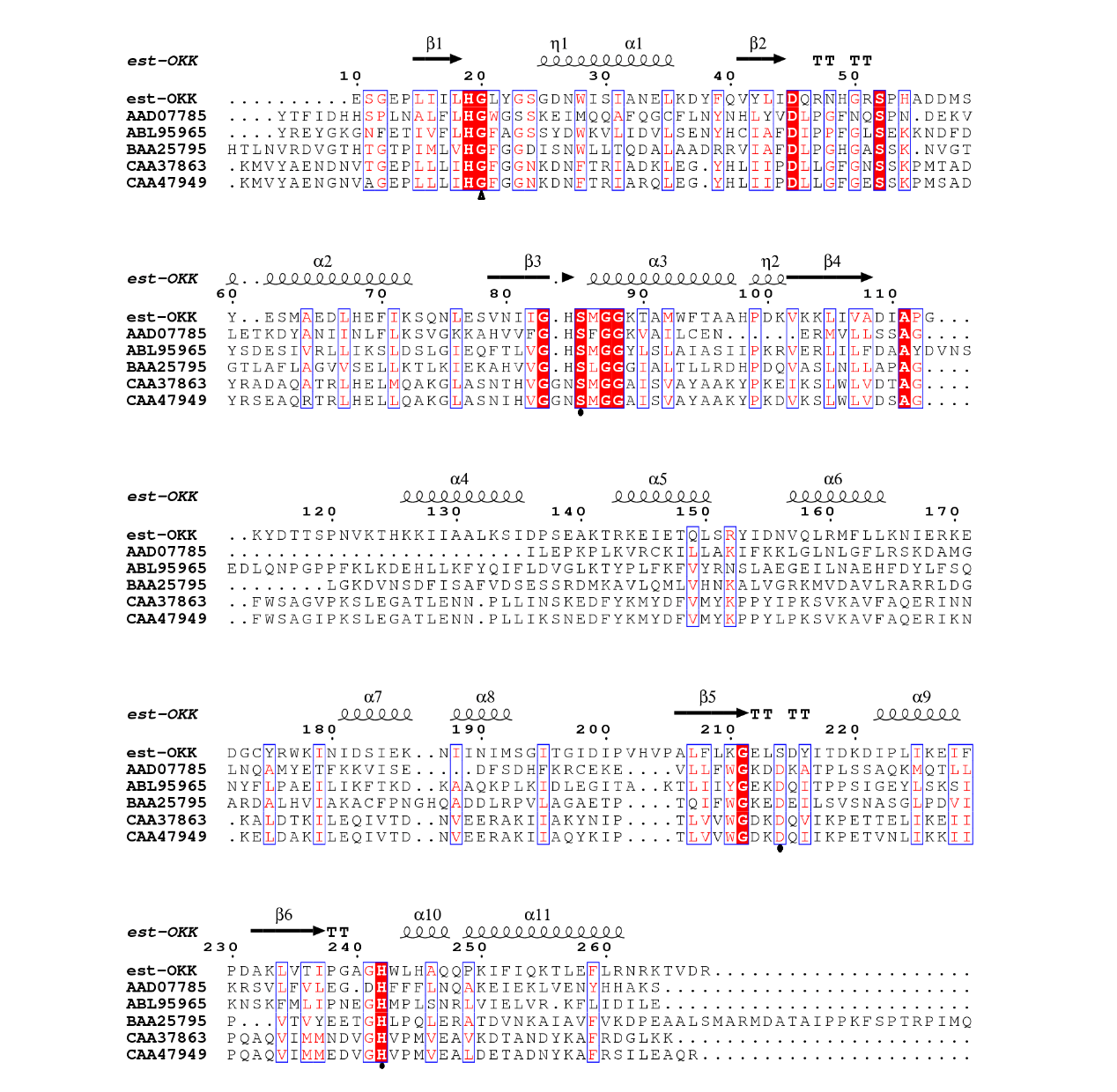


**Figure S2** Alignment of the amino acid sequences of family V lipolytic enzymes CAA47949 (*Psychrobacter immobilis*), CAA37863 (*Moraxella* sp.), ABL95965 (*Fervidobacterium changbaicum*), BAA25795 (*Acetobacter pasteurianus*), and AAD07785 (*Helicobacter pylori*). A putative oxyanion hole and the active-site residues are indicated by a triangle and circles below the sequences, respectively. Similar residues are indicated by a clear box, and identical residues are indicated by a colored background.


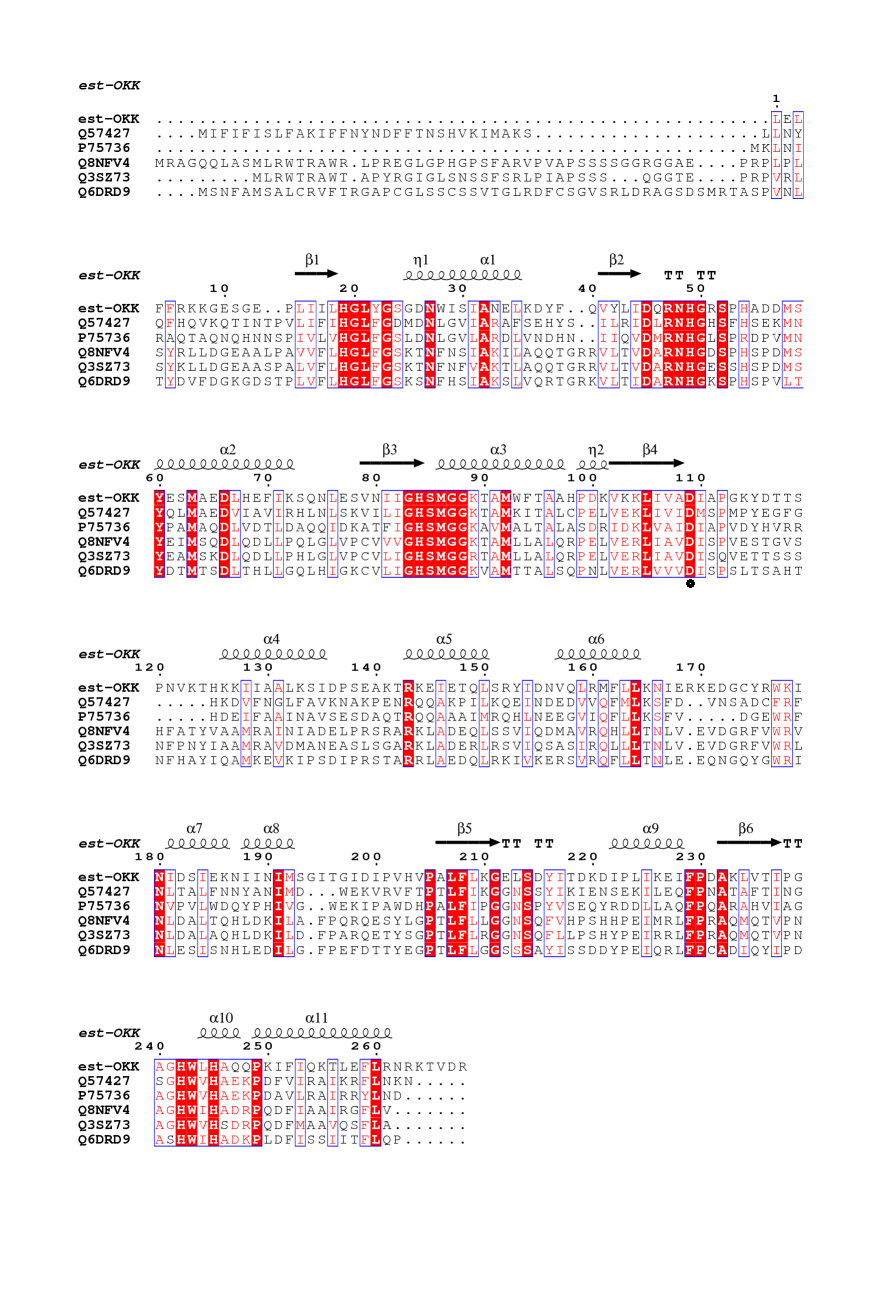


**Figure S3** Sequence alignments were performed using the CLUSTALW and ESPript3.0 programs. The additional conserved site Asp109 was indicated by a circles. Similar residues are indicated by a clear box, and identical residues are indicated by a colored background.

**
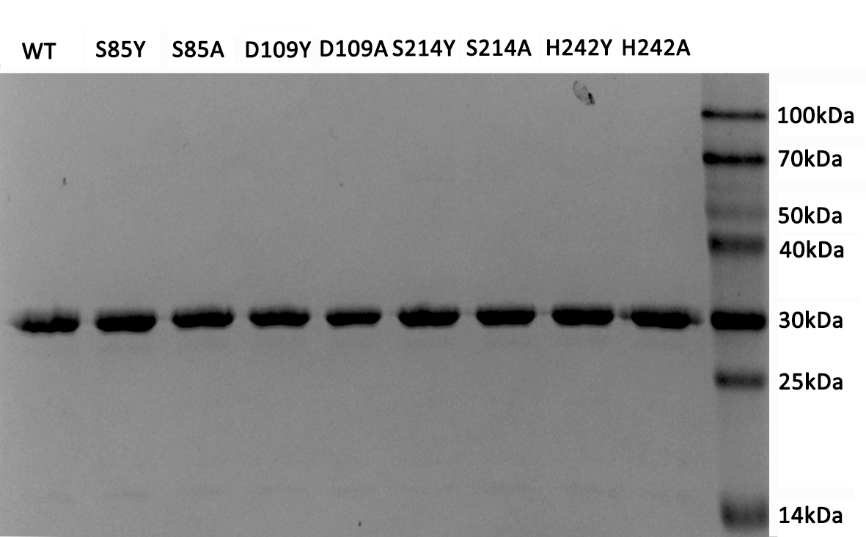
**

**Figure S4** SDS-PAGE analysis of est-OKK proteins from WT and variants (S85Y, S85A, D109Y, D109A, S214Y, S214A, H242Y and H242A) after Ni affinity chromatography (S85Y represents the eighty-fifth serine mutation into tyrosine of est-OKK; Ser, Asp, His, Ala and Tyr were represented by S, D, H, A and Y).





**Figure S5** Effect of the concentrations of p-NP-butyrate on velocity of est-OKK at pH 7.5 and 50°C; fitted curve based on the Michaelis-Menten equation.


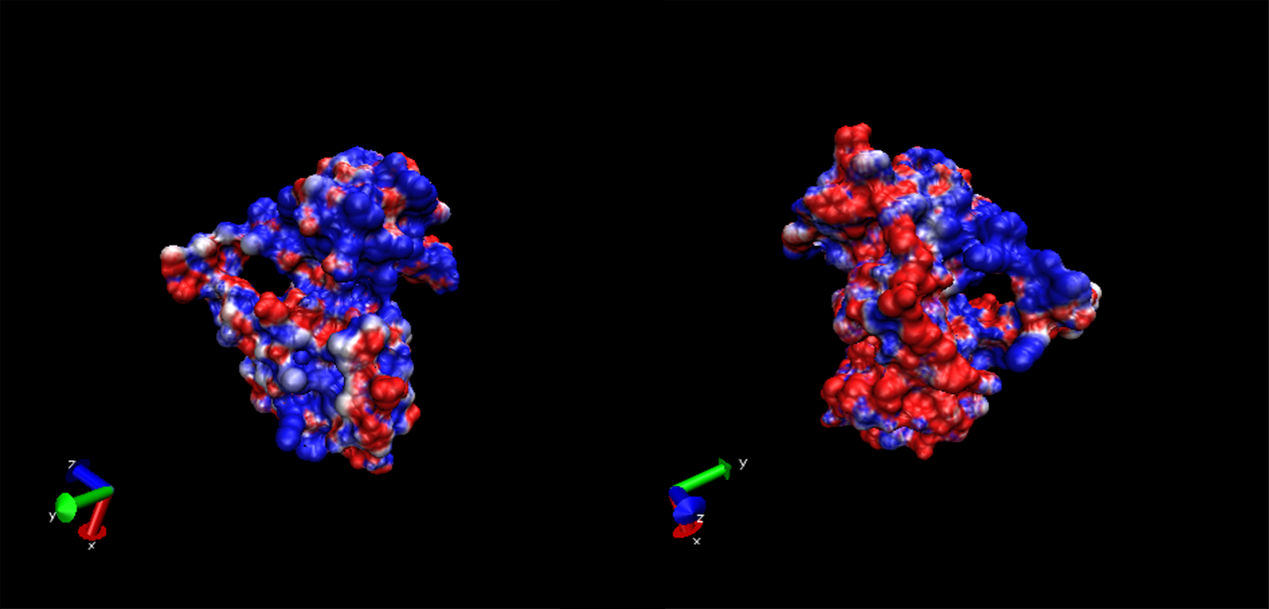


**Figure S6** The surface electrostatic potential of est-OKK obtained by VMD and APBS plugins. The negative and positive electrostatic potentials are indicated by blue and red, respectively.

**References**

[1] De, S. C., Leiros, H. K., Di, S. A., De, P. D., Altermark, B. and Willassen, N. P. (2016). Biochemical characterization and structural analysis of a new cold-active and salt-tolerant esterase from the marine bacterium *Thalassospira* sp. *Extremophiles* **20**, 323-336.

[2] Jeon, J. H., Lee, H. S., Kim, J. T., Kim, S.-J., Choi, S. H., Kang, S. G. and Lee, J.-H. (2011). Identification of a new subfamily of salt-tolerant esterases from a metagenomic library of tidal flat sediment. *Appl Microbiol Biotechnol* **93**, 623-631.

[3] Rodriguez, M. C., Loaces, I., Amarelle, V., Senatore, D., Iriarte, A., Fabiano, E. and Noya, F. (2015). Est10: A Novel Alkaline Esterase Isolated from Bovine Rumen Belonging to the New Family XV of Lipolytic Enzymes. *PLoS One* **10**, e0126651.

[4] Wu, G., Zhang, S., Zhang, H., Zhang, S. and Liu, Z. (2013). A novel esterase from a psychrotrophic bacterium *Psychrobacter celer* 3Pb1 showed cold-adaptation and salt-tolerance. J Mol Catal B-Enzym **98**, 119-126.

[5] Zhang, Y., Hao, J., Zhang, Y. Q., Chen, X. L., Xie, B. B., Shi, M. and Li, P. Y. (2017). Identification and Characterization of a Novel Salt-Tolerant Esterase from the Deep-Sea Sediment of the South China Sea. *Front Microbiol* **8**, 441.
